# Phylogeny of Muhlenbergia subg. Pseudosporobolus, including *M. spatha* (Poaceae, Chloridoideae, Cynodonteae, Muhlenbergiinae) now found in Zacatecas, Mexico

**DOI:** 10.3897/phytokeys.103.26162

**Published:** 2018-07-18

**Authors:** Paul M. Peterson, Yolanda Herrera Arrieta, Konstantin Romaschenko

**Affiliations:** 1 Department of Botany MRC-166, National Museum of Natural History, Smithsonian Institution, Washington, DC 20013-7012, USA; 2 Instituto Politécnico Nacional, CIIDIR Unidad Durango-COFAA, Durango C.P. 34220, México

**Keywords:** grasses, ITS, Mexico, *Muhlenbergia*, plastid DNA sequences, *Schaffnerella gracilis*, systematics, taxonomy

## Abstract

*Muhlenbergia spatha*, previously known only from near the type locality in San Luis Potosí, is reported from two localities in Zacatecas, Mexico. Historically, botanists have overlooked this diminutive annual. To clarify affinities of *M. spatha*, we present a molecular phylogeny emphasising species in M. subg. Pseudosporobolus using sequence data from two plastid markers (*rpl32-trnL* and *rps16* intron) and nrDNA ITS. In addition, we include an updated description, illustration and discussion of the habitat of *M. spatha*.

## Abstract (es)

*Muhlenbergia spatha*, anteriormente conocida solo cerca de la localidad tipo en San Luis Potosí, se reporta en dos localidades en Zacatecas, México. Históricamente, los botánicos han pasado por alto esta diminuta anual. Para aclarar las afinidades de *M. spatha*, presentamos una filogenia molecular que enfatiza especies en M. subg. Pseudosporobolus usando datos de secuencia de dos marcadores plástidos (*rpl32-trnL* e *rps16* intron) y nrADN EIT. Además, incluimos una descripción actualizada, ilustración y discusión del hábitat de *M. spatha*.

## Introduction

*Muhlenbergia spatha* Columbus is a small (usually less than 20 cm tall) annual first collected in the mountains of San Miguelito, in the valley of San Luis Potosí by J.G. Schaffner in 1876 (Bentham 1882). There are few collections of this species (only two known numbers collected by Schaffner with at least 14 duplicates) and it was thought to be extirpated until it was rediscovered near the type locality by J.T. Columbus along the Río Potosino in the Sierra de San Miguelito in 2001 (Columbus et al. 2001). *Muhlenbergia spatha* can be separated from other species in the genus by its possessing small, few-branched condensed panicles that are partially included in a spatheolate sheath with each branch containing 1–5 sessile spikelets.

Bentham (1882) thought *Schaffnerella gracilis* (Benth.) Nash (≡ *Schaffnera gracilis* Benth. ≡ *Muhlenbergia spatha*) was related to other members of the Zoysieae (e.g. *Zoysia* Willd. and *Aegopogon* P. Beauv = *Muhlenbergia* Schreb.), but he also suggested the inflorescences were nearer to members of the Andropogoneae or Pappophoreae. Beal (1896), Nash (1912), and Conzatti (1988) followed Bentham and included the monotypic *Schaffnerella* Nash in the Zoysieae. There are no modern treatments of *Schaffnerella* in Mexico, although a fairly complete genus and species description is given in Nash (1912) and, later, much abbreviated in Conzatti (1988). Pilger (1956) placed *Schaffnerella* in the tribe Lappagineae Link, again near *Zoysia* and *Aegopogon*. Clayton and Renvoize (1986) indicated *Schaffnerella* was “an isolated genus apparently related to *Opizia*” J. Presl (= *Bouteloua* Lag.; Peterson et al. 2015) and placed it in the tribe Cynodonteae. Currently, the Cynodonteae includes 25 subtribes, 95 genera and 859 species (Peterson et al. 2010a, 2016, 2017; Soreng et al. 2017). Molecular DNA sequence studies support placement of *Schaffnerella* in the Cynodonteae, aligning the genus within the monogeneric subtribe Muhlenbergiinae Pilg. in Muhlenbergia Schreb. subg. Pseudosporobolus (Parodi) P.M. Peterson (Columbus et al. 2010; Peterson et al. 2010a, 2010b). There are at least 27 species of *Muhlenbergia* that align within M. subg. Pseudosporobolus.

In this paper, we report two new collections of *Muhlenbergia spatha* in Zacatecas, include a complete updated description and illustration and present a molecular phylogeny of species in M. subg. Pseudosporobolus using sequence data from two plastid markers (*rpl32-trnL* and *rps16* intron) and a single nuclear marker (ITS). Our new molecular phylogeny of M. subg. Pseudosporobolus was included to characterise evolutionary relationships of *M. spatha*, since earlier analyses for this species were based on few markers (Columbus et al. 2010; Peterson et al. 2010b). Based on a single sequence (*rpl32-trnL*), *M. spatha* paired with *M. alopecuroides* (Griseb.) P.M. Peterson & Columbus in a plastid tree and it paired with *M. wrightii* Vasey ex J.M. Coult. in a combined (plastid and ITS) tree (Peterson et al. 2010b).

## Materials and methods

**Phylogenetic analyses**. Detailed methods for DNA extraction, amplification, sequencing and phylogenetic analysis are given in Peterson et al. (2010a, 2010b, 2014, 2015, 2016). In brief, we estimated the phylogeny amongst members of *Muhlenbergia* based on the analysis of three molecular markers (nuclear ribosomal ITS 1&2; plastid *rpl32-trnL* and *rps16* intron DNA sequences). For this study we included a sampling of species within the five subgenera of *Muhlenbergia*, *M. ramulosa* (Kunth) Swallen and the outgroups *Distichlis scoparia* (Nees ex Kunth) Arechav. (Cynodonteae, Monanthochloinae), *Sporobolus indicus* (L.) R. Br. (Zoysieae, Sporobolinae) and *Willkommia sarmentosa* Hack. (Cynodonteae, Traginae) [Peterson et al. 2010b, 2016]. Voucher information and GenBank numbers for all 41 samples used in the analysis are given in Table 1.

**Table 1.** Taxon voucher (collector, number and where the specimen is housed), country of origin and GenBank accession for DNA sequences of rps16 intron, rpl32-trnL and ITS regions (**bold** indicates new accession); a dash (–) indicates missing data.

N	Taxon	Voucher	Country	rps16 intron	rpl32-trnL	ITS
	**OUTGROUP**					
1	Distichlis scoparia var. erinacea (Nees ex Kunth) Arechav.	Peterson 17475, Soreng & Refulio-Rodriguez (US)	Argentina, Neuquen	GU360477	GU359803	GU359334
2	*Sporobolus indicus* (L.) R. Br.	Peterson 22025 & Saarela (US)	Mexico, Chihuahua	GU360355	GU359913	GU359209
3	*Willkommia sarmentosa* Hack.	Schweickerdt 2181 (US)	South Africa,	GU360343	GU359924	GU359194
	**MUHLENBERGIA**					
4	*Muhlenbergia ramulosa* (Kunth) Swallen	Peterson 22447 & Saarela (US)	Mexico, Durango	GU360406	GU359953	GU359115
	**subg. Bealia**					
5	*Muhlenbergia arenicola* Buckley	Peterson 19947 & Lara-Contreras (US)	Mexico, Coahuila	GU360413	GU359960	GU359166
6	*Muhlenbergia tricholepis* (Torr.) Columbus	Peterson 22099 & Saarela (US)	Mexico, Chihuahua	GU360305	GU359853	GU359278
	**subg. Clomena**					
7	*Muhlenbergia durangensis* Y. Herrera	Peterson 13644, Knowles, Dietrich, Braxton & Gonzalez-Elizondo (US)	Mexico, Durango	HM143552	HM143162	HM143060
8	*Muhlenbergia montana* (Nutt.) Hitchc.	Peterson 22234 & Saarela (US)	Mexico, Sinaloa	GU360417	GU359964	GU359162
	**subg. Muhlenbergia**					
9	*Muhlenbergia glauca* (Nees) B.D. Jacks.	Peterson 21023, Saarela, Lara Contreras & Reyna Alvarez (US)	Mexico, Coahuila	HM143563	HM143173	HM143072
10	*Muhlenbergia pereilema* P.M. Peterson	Peterson 22191 & Saarela (US)	Mexico, Sinaloa	GU360282	GU359993	GU359131
	**subg. Pseudosporobolus**					
11	*Muhlenbergia alopecuroides* (Griseb.) P.M. Peterson & Columbus	Peterson 20960, Saarela, Lara Contreras & Reyna Alvarez (US)	Mexico,	GU360426	GU359976	GU359152
12	*Muhlenbergia alopecuroides* (Griseb.) P.M. Peterson & Columbus	Peterson 22008 & Saarela (US)	Mexico, Chihuahua	GU360425	GU359975	GU359153
13	*Muhlenbergia arenacea* (Buckley) Hitchc.	Peterson 10624 & Annable (US)	Mexico, Coahuila	GU360414	GU359961	GU359165
14	*Muhlenbergia asperifolia* (Nees & Meyen ex Trin.) Parodi	Peterson 15452, Soreng, Finot & Judziewicz (US)	Chile, Region III (Atacama)	HM143539	HM143149	HM143048
15	*Muhlenbergia atacamensis* Parodi	Peterson 19626, Soreng, Salariato, & Panizza, (US)	Argentina, Jujuy	GU360489	GU359879	GU359344
16	*Muhlenbergia cuspidata* (Torr. ex Hook.) Rydb.	Hill 35331 (US)	USA	HM143546	HM143156	HM143055
17	*Muhlenbergia decumbens* Swallen	Columbus 3653 (RSA)	Mexico	–	–	EF153029
18	*Muhlenbergia fastigiata* (J. Presl) Henrard	Peterson 21512, Soreng, LaTorre & Rojas Fox (US)	Peru, Ancash	HM143556	HM143166	HM143064
19	*Muhlenbergia implicata* (Kunth) Trin.	Peterson 22266, Saarela (US)	Mexico, Oaxaca	HM143568	HM143179	HM143077
20	*Muhlenbergia jaime-hintonii* P.M. Peterson & Valdés-Reyna	Peterson 15841 & Valdés-Reyna (US)	Mexico, Nuevo León	HM143569	HM143181	HM143079
21	*Muhlenbergia ligulata* (E. Fourn.) Scribn. & Merr.	Peterson 22416 & Saarela (US)	Mexico, Durango	GU360440	GU359863	GU359273
22	*Muhlenbergia monandra* Alegría & Rúgolo	Peterson 17990 & Refulio-Rodriguez (US)	Peru, Lima	–	–	GQ397891
23	*Muhlenbergia multiflora* Columbus	Peterson 7845 & Annable (US)	USA, Colorado	GU360289	GU359985	GU359138
24	*Muhlenbergia palmirensis* Grignon & Lægaard	Peterson 9317 & Judziewicz (US)	Ecuador, Chimborazo	HM143586	HM143200	HM143098
25	*Muhlenbergia paniculata* (Nutt.) Columbus	Peterson 12070 & Annable (US)	USA, Colorado	GU360375	GU359936	GU359201
26	*Muhlenbergia phleoides* (Kunth) Columbus	Peterson 24452, Romaschenko & Valdés-Reyna (US)	Mexico, Nuevo León	–	**MH400231**	**MH400228**
27	*Muhlenbergia phleoides* (Kunth) Columbus	Peterson 24799, Romaschenko, Rodriguez Avalos, Herrera-Simoni, & Garcia Rodriguez (US)	Mexico, Aguascalientes	–	**MH400232**	**MH400229**
28	*Muhlenbergia pungens* Thurb. ex A. Gray	Ricketson 4642 (MO)	USA, Arizona	**MH508106**	**MH508102**	**MH508098**
29	*Muhlenbergia repens* (J. Presl) Hitchc.	Peterson 7900 & Annable (US)	USA, New Mexico	**HM143596**	**HM143212**	**HM143110**
30	*Muhlenbergia richardsonis* (Trin.) Rydb.	Peterson 7832 & Annable (US)	USA, Colorado.	HM143598	HM143214	HM143112
31	*Muhlenbergia seatonii* Scribn.	Peterson 9946	Mexico, Puebla	**MH508107**	**MH508103**	**MH508099**
32	*Muhlenbergia spatha* Columbus	Schaffner 134 (US)	Mexico,	–	GU359981	**MH400230**
33	*Muhlenbergia subbiflora* Hitchc.	Peterson 21158, Saarela, Rosen & Reid (US)	Mexico, Durango	GU360439	GU359877	GU359318
34	*Muhlenbergia tenuissima* (J. Presl) Kunth	Peterson 4751 & Annable	Mexico, Jalisco	**MH508108**	**MH508104**	**MH508100**
35	*Muhlenbergia uniflora* (Muhl.) Fernald	Peterson 13212, Annable, Pizzolato, Gordon, Frett, Frick, Morrone & Griner (US)	USA, New Jersey	HM143616	HM143232	HM143130
36	*Muhlenbergia uniflora* (Muhl.) Fernald	Peterson 20862 & Saarela (US)	USA, New York	GU360275	GU359994	GU359119
37	*Muhlenbergia utilis* (Torr.) Hitchc.	Peterson 24869 & Romaschenko (US)	Mexico, San Luis Potosí	–	**MH508105**	**MH508101**
38	*Muhlenbergia villiflora* Hitchc.	Peterson 15811 & Valdés-Reyna (US)	Mexico, Nuevo León	HM143620	HM143236	HM143133
39	*Muhlenbergia wrightii* Vasey ex J.M. Coult.	Peterson 20964, Saarela, Lara Contreras & Reyna Alvarez (US)	Mexico, Coahuila	HM143623	HM143240	HM143137
	**subg. Trichochloa**					
40	*Muhlenbergia rigens* (Benth.) Hitchc.	Peterson 22129 & Saarela (US)	Mexico, Chihuahua	GU360357	GU359951	GU359117
41	*Muhlenbergia longiligula* Hitchc.	Peterson 15224 & Cayouette (US)	USA, Arizona	HM143574	HM143187	HM143085

## Results and discussion

**Figure 1.**
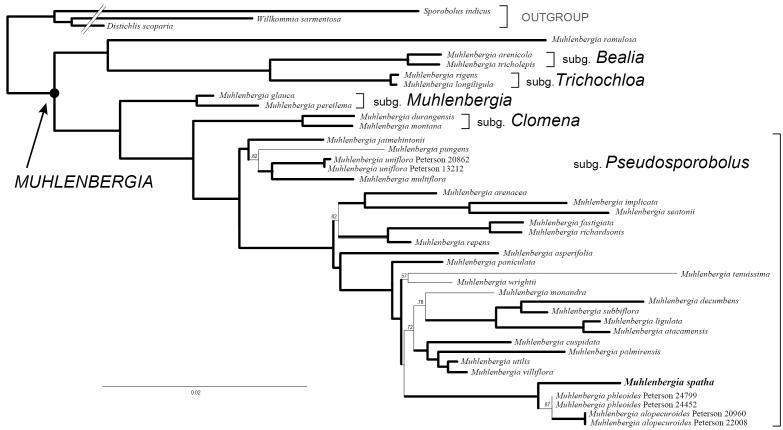
Maximum-likelihood tree inferred from combined plastid (*rpl32-trnL, rps16* intron) and ITS sequences. Thick branches indicate posterior probabilities of 1; numbers above branches are posterior probabilities less than 1 but greater than 0.50; Scale bar: 2%.

**Phylogeny.** A total of 16 new sequences from *Muhlenbergia phleoides* (Kunth) Columbus, *M. pungens* Thurb. ex A. Gray, *M. seatonii* Scribn., *M. spatha, M. tenuissima* (J. Presl) Kunth and *M. utilis* (Torr.) Hitchc. are reported in GenBank (Table 1). Total aligned characters for individual regions and other parameters are noted in Table 2. In Figure 1, we combined the plastid–ITS sequences in our analysis since there was little incongruence between these data sets.

**Table 2.** Characteristics of the three regions, *rpL32-trnL*, *rps16* intron and ITS and parameters used in Bayesian analyses indicated by Akaike Information Criterion (AIC).

	*rpL32-trnL*	*rps16 intron*	*Combined plastid data*	*ITS*	*Overall*
Total aligned characters	996	1088	2084	761	2845
Likelihood score (-lnL)	4758.44	3429.94		9569.67	
Number of substitution types	6	6	−	6	−
Model for amongst-site rate variation	gamma	gamma	−	gamma	−
Substitution rates					
rAC	1.1071	1.2315	−	1.5168	−
rAG	1.8768	1.2968		2.8806	
rAT	0.4688	0.4669		1.9244	
rCG	1.2702	1.0243		0.7054	
rCT	1.4748	2.4545		5.3115	
rGT	1.0000	1.0000		1.0000	
Character state frequencies					
fA	0.3827	0.3860	−	0.2585	−
fC	0.1189	0.1105		0.1967	
fG	0.1241	0.1772		0.2539	
fT	0.3740	0.3261		0.2907	
Proportion of invariable sites	0.1608	0.3844	−	0.3790	−
Substitution model	TVM+G	TVM+G	−	GTR+I+G	−
Gamma shape parameter (α)	0.9290	0.9303	−	0.7988	−

The maximum-likelihood tree from the combined analysis of ITS and two plastid regions (*rpL32-trnL* and *rps16* intron) is well resolved, with strong support for the monophyly of *Muhlenbergia*, including *M. ramulosa* and five subgenera, *Bealia*, *Clomena*, *Muhlenbergia*, *Pseudosporobolus* and *Trichochloa* [Fig. 1; posterior probability (PP) = 1 for thick branches]. Within Muhlenbergia subg. Pseudosporobolus, *M. spatha* is strongly supported (PP = 1) as sister to *M. phleoides* and *M. alopecuroides*. *Muhlenbergia decumbens* Swallen, *M. monandra* Alegría & Rúgolo, *M. pungens*, *M. seatonii* and *M. tenuissima* are newly reported as occurring in M. subg. Pseudosporobolus, bringing the total to 27 species for this subgenus.

It is interesting but not surprising that *Muhlenbergia phleoides*, a close relative of *M. spatha*, was found as an associated species at both collection sites in Zacatecas (*Rosales 3490 & Herrera Arrieta*; *Peterson 25544 & Herrera Arrieta*). *Muhlenbergia spatha*, *M. alopecuroides*, *M. phleoides* and *M. phalaroides* Kunth (a presumed member of this clade, not yet sampled and not included in our tree) all have plumbeous-mottled spikelets that disarticulate as a unit (below the glumes) leaving a small cuplike tip and glumes with 2–5 recurved awns (Reeder 2003; Peterson et al. in prep.). Plumbeous-mottled spikelets and well-developed sclerenchyma girders in the primary vascular bundles are additional traits shared by members of M. subg. Pseudosporobolus (Watson and Dallwitz 1992; Peterson and Herrera Arrieta 2001; Peterson et al. 2010b).

## Taxonomy

### Muhlenbergia spatha

Taxon classificationPlantaePoalesPoaceae

Columbus, Aliso 28: 66. 2010, non Muhlenbergia gracilis (Kunth) Trin. (1824).

Fig. 2A–D

Schaffnera gracilis Benth., Hooker’s Icon. Pl. 14: 59, t. 1378. 1882. Schaffnerella gracilis (Benth.) Nash, N. Amer. Fl. 17(2): 141. 1912, non Schaffneria Fée ex T. Moore (1857). Muhlenbergia columbi P.M. Peterson, Amer. J. Bot. 97(9): 1543. 2010, nom. illeg. superfl. Type: Mexico, San Luis Potosí, mountains of San Miguelita, Aug 1876, *J.G. Schaffner 1070* (holotype: K-000309066 [image!]; isotypes PH-00022592 [image!], US-397116!, YU-063983 [image!]).

#### Description.

Delicate **annuals**, loosely caespitose. **Culms** 5–20(–30) cm tall, erect, geniculate below; **nodes** scaberulous, branching at lower and middle nodes; **internodes** 2.0–4.5(–8.0) cm long, strongly 4–6 ribbed. **Leaves** caulescent and basal; **ligules** 0.8–1.8 mm long, membranous, decurrent, apex obtuse, minutely erose; **sheaths** 0.8–1.5 cm long, much shorter than the internodes, oblong, open, chartaceous, strongly ribbed with 7–9-veins, sometimes keeled, margins hyaline; **blades** 0.5–4 cm long, 0.5–1.5 mm wide, flat or folded, adaxially scattered pubescent near base, the hairs antrorse leaning, apex obtuse. **Inflorescences** compound, fasciculate, composed of terminal and axillary condensed **panicles**, these branched near the base, the basal-most branch usually with a sterile floret at the base consisting of two scales, the entire panicle partially included in a spatheolate sheath; **sterile florets** 2–4 mm long, 0.8–1 mm wide, linear-apiculate, flat, hyaline, 1-veined; **racemose branches** each bearing 1–5 fertile sessile spikelets, the spikelets separated by 1–4 mm on each branch; **rachis** angular, 3 or 4-ribbed. **Spikelets** 4.4–6 mm long, lanceolate, laterally compressed, solitary, composed of one fertile floret, plumbeous-mottled; **rachilla** not extended; **callus** short, blunt, pubescent, located just below the glumes where disarticulation occurs leaving a small cuplike tip; **glumes** dimorphic; **lower glumes** absent or obscure; **upper glumes** 3.5–5(–6) mm long, about as long as the lemma, oblong, chartaceous, firmer than fertile lemma (excluding the awns), 7–9-veined, lateral veins ribbed, pubescent along the veins on lower ½, apex deeply bifid and 3(5)–awned, the awns 5–7 mm long, scabrous, recurved, arising between the bifid apex; **lemmas** (4–)4.8–6 mm long, lanceolate, membranous, 3-veined, keeled, midvein scaberulous, lateral veins ribbed, apex acute to acuminate, minutely bifid, 1-awned, the awn 3–5 mm long arising from between the teeth; **paleas** 3.7–5.5 mm long, shorter than the lemmas, oblong, hyaline to membranous, tightly involute, 2-veined, apex obtuse, unawned; **stamens** 3; **anthers** 2–2.5 mm long, yellowish; **ovary** glabrous; **caryopses** 1.8–2 mm long, 0.5 mm wide, narrowly fusiform, straw coloured.

**Figure 2.**
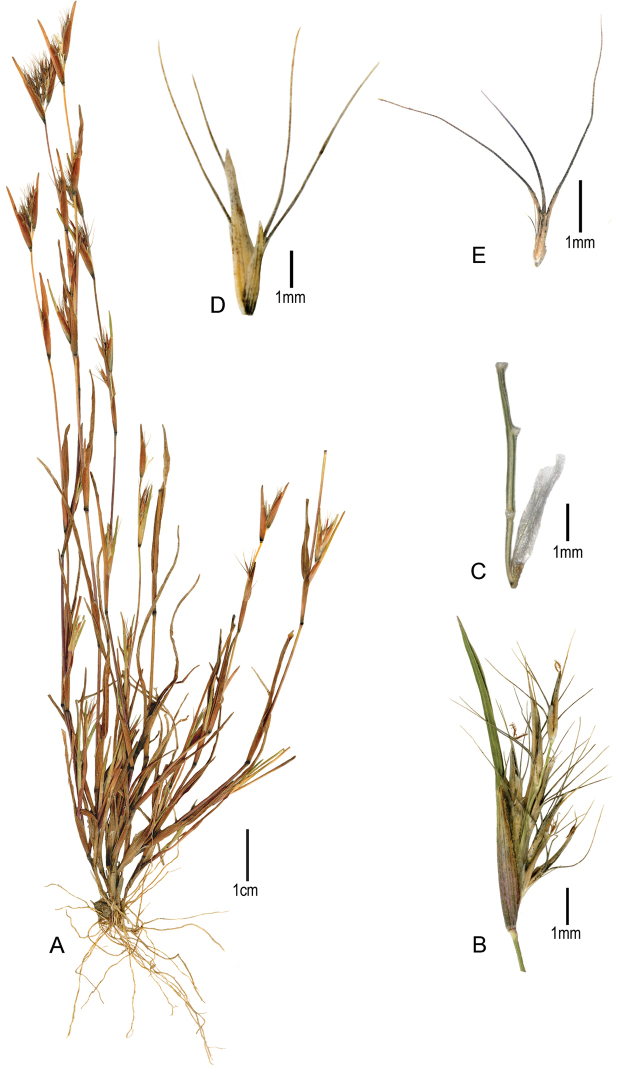
*Muhlenbergia spatha*: **A** Habit **B** Panicle partially enclosed by the leaf sheath **C** Basal panicle branch with a lower sterile floret and small cuplike structures (callus remains) where the fertile spikelets were inserted **D** Spikelet, side view **E** Upper glume showing three recurved awns, dorsal view. (*Peterson 25544 & Herrera Arrieta*, CIIDIR).

#### Distribution.

The species is known only from the type locality in San Luis Potosí and Municipio Villanueva, Zacatecas.

#### Ecology.

*Muhlenbergia spatha* was found by the authors growing on flat table rock in open areas near arroyos associated with *Bouteloua hirsuta* Lag., *B. curtipendula* (Michx.) Torr., *Schizachyrium sanguineum* (Retz.) Alston, *Quercus*, *Juniperus*, *Muhlenbergia implicata* (Kunth) Trin., *M. phleoides*, *M. rigida* (Kunth) Kunth, *Chloris submutica* Kunth, *Digitaria ternata* (A. Rich.) Stapf, *Microchloa kunthii* Desv., *Aristida adscensionis* L., *A. divaricata* Humb. & Bonpl. ex Willd., *Enneapogon desvauxii* P. Beauv., *Piptochaetium fimbriatum* (Kunth) Hitchc., *Eragrostis intermedia* Hitchc., *E. pectincea* (Michx.) Nees and *Eleusine multiflora* Hochst. ex A. Rich.

#### Habitat.

At the southern edge of the Sierra Madre Occidental Range in the Sierra Fría de Aguascalientes, we recently found *Muhlenbergia spatha* in two localities south of the city of Zacatecas in a corridor located east of Villanueva: 1) 17.4 km east of Villanueva on flat table rock just above Arroyo “El Muerto” in open areas and 2) about 1 km northeast of the small village Palomas Viejas along Arroyo Juan Manuel on natural grasslands near cultivated fields. These two sites are approximately 204 km W (air distance) from the type locality southwest of San Luis Potosí in the Sierra de San Miguelito along the Río Potosino (Columbus et al. 2001). According to Instituto Nacional de Estadística, Geografía e Informática [INEGI] (2003, 2005), the Sierra San Miguelito is placed in the Mesa del Centro Province in the Sierra Madre Oriental. Near Villanueva, Zacatecas, the soils are Haplic Phaeozem (forming sodium carbonate, Na_2_CO_3_) over extrusive igneous rock (acidic) in a semi-dry climate with an annual mean temperature of 16°C and an annual mean precipitation of 60 cm (INEGI 2008a, 2008b). In the Sierra de San Miguelito along the Rio Potosino, the soils are Calcic Regosol (forming calcium carbonate, CaCO_3_) of medium texture over extrusive igneous rock (acidic) in a semi-dry climate with an annual mean temperature of 16 °C and an annual mean precipitation of 40 cm (INEGI 2008a, 2008b). Without field verification, it is uncertain whether the soil differences among these sites are significant, but we are reasonably certain that all are alkaline with an elevated pH.

#### Conservation status.

The species is rare in Mexico and is known from only three recent collections. Since it is a diminutive, short-lived annual, the species is easily overlooked and the main concern seems to be loss of habitat via human impact, i.e. agriculture, dam and road construction.

#### Specimens examined.

Mexico. **San Luis Potosí**: 1876, *J.G. Schaffner 134* (GOET-006918 [image!], US-825687); Sierra de San Miguelito, Río Potosino, 22°04'55"N, 101°03'51"W, 1980 m, 2 Oct 2001, *J.T. Columbus 4040* (RSA [image! in Columbus et al. 2001]). **Zacatecas**: Mpio. Villanueva, Arroyo Juan Manuel ± 1 km NE of Palomas Viejas near Villanueva, 22°24'36"N, 102°43'01"W, 2112 m, 22 Oct 2006, *O. Rosales 3490 & Y. Herrera Arrieta* (CIIDIR); Mpio Villanueva, 10.8 mi [17.4 km] E of Villanueva, just above arroyo “El Muerto”, 22°22'47.1"N, 102°43'31.5"W, 2083 m, 2 Oct 2015, *P.M. Peterson 25544 & Y. Herrera Arrieta* (CIIDIR, US).

#### Comments.

The hand written script (verified by J. Rzedowski, per. comm., also see Rzedowski 1959) on the label of the holotype (K) says: “*Müehlenbergia gracilis mihi*”, indicating that Jose Guillermo Schaffner thought he had collected a new species of *Muhlenbergia*. Bentham (1882) agreed with Schaffner that it was a new species but thought it had enough unique morphological features (spatheolate sheathed panicles, sessile spikelets and 3(5)-awned upper glumes) to warrant description of a new genus.

## Supplementary Material

XML Treatment for Muhlenbergia spatha

## References

[B1] Beal WJ (1896) Grasses of North America, Vol. II. Henry Holt and Company, New York, 1–706.

[B2] Bentham G (1882) Plate 1378. *Schaffnera gracilis*, Benth. Gramineae, Tribe Zoysieae? Hooker’s Icones Plantarum 14: 59.

[B3] Clayton WD Renvoize SA (1986) Genera graminum. Grasses of the world. Kew Bulletin, Additional Series 13: 1–389.

[B4] Columbus JT Bell HL Cerros-Tlatilpa R Griffith MP Porter JM (2001) *Schaffnerella* rediscovered! (Gramineae, Chloridoideae). Aliso 20: 45–90. 10.5642/aliso.20012001.08

[B5] Columbus JT Peterson PM Refulio Rodríguez NF Cerros-Tlatilpa R Kinney MS (2010) Phylogenetics of Muhlenbergiinae (Poaceae: Chloridoideae, Cynodonteae) based on ITS and *trnL-F* DNA sequences. Aarhus University Press, Aarhus, 477–496.

[B6] Conzatti C (1988) Flora taxonomica Mexicana, Vol. I. Consejo Nacional de Ciencia Y Tecnologia, Mexico D.F., 1064 pp.

[B7] INEGI (2003) Cartas de Uso del Suelo y Vegetación. Conjunto de datos vectoriales de la carta de Uso del suelo y vegetación. Escala 1:1 000000. Serie II (Continuo Nacional). http://www.inegi.org.mx/

[B8] INEGI (2005) Geología. Conjunto de datos vectoriales. Escala 1:1 000000. http://www.inegi.org.mx/

[B9] INEGI (2008a) Cartas climatológicas. Conjunto de datos vectoriales. Escala 1:1 000000. http://www.inegi.org.mx/

[B10] INEGI (2008b) Perfiles de Suelos, Estados Unidos Mexicanos. Conjunto de datos vectoriales. Escala 1:1000000. http://www.inegi.org.mx/

[B11] Nash GV (1912) 36. *Schaffnerella* Nash. In: Britton NL, Murrill WA, Barnhart JH (Eds) North American flora 17(2). New York Botanical Garden, New York, 141.

[B12] Peterson PM Herrera Arrieta Y (2001) A leaf blade anatomical survey of *Muhlenbergia* (Poaceae: Muhlenbergiinae). Sida 19: 469–506.

[B13] Peterson PM Romaschenko K Herrera Arrieta Y (2015) Phylogeny and subgeneric classification of *Bouteloua* with a new species, *B. herrera-arrietae* (Poaceae: Chloridoideae: Cynodonteae: Boutelouinae). Journal of Systematics and Evolution 53(4): 351–366. https://onlinelibrary.wiley.com/doi/epdf/10.1111/jse.12159. 10.1111/jse.12159

[B14] Peterson PM Romaschenko K Herrera Arrieta Y (2016) A molecular phylogeny and classification of the Cynodonteae (Poaceae: Chloridoideae) with four new genera: *Orthacanthus*, *Triplasiella*, *Tripogonella*, and *Zaqiqah*; three new subtribes: Dactylocteniinae, Orininae, and Zaqiqahinae; and a subgeneric classification of *Distichlis*. Taxon 65(6): 1263–1287. 10.12705/656.4

[B15] Peterson PM Romaschenko K Herrera Arrieta Y (2017) Four new subtribes: Allolepiinae, Jouveinae, Kaliniinae, and Sohnsiinae in the Cynodonteae (Poaceae: Chloridoideae). Phytoneuron 2017–44: 1–9. http://www.phytoneuron.net/2017Phytoneuron/44PhytoN-ChloridoidSubtribes.pdf

[B16] Peterson PM Romaschenko K Johnson G (2010a) A classification of the Chloridoideae (Poaceae) based on multi-gene phylogenetic trees. Molecular Phylogenetics and Evolution 55(2): 580–598. 10.1016/j.ympev.2010.01.018 PubMed 20096795

[B17] Peterson PM Romaschenko K Johnson G (2010b) A phylogeny and classification of the Muhlenbergiinae (Poaceae: Chloridoideae: Cynodonteae) based on plastid and nuclear DNA sequences. American Journal of Botany 97(9): 1532–1554. 10.3732/ajb.0900359 PubMed 21616906

[B18] Peterson PM Romaschenko K Soreng RJ (2014) A laboratory guide for generating DNA barcodes in grasses: a case study of *Leptochloa* s.l. (Poaceae: Chloridoideae). Webbia 69(1): 1–12. 10.1080/00837792.2014.927555

[B19] Pilger R (1956) Gramineae II. Duncker & Humblot, Berlin, 1–168.

[B20] Reeder CG (2003) 17.34 *Lycurus* Kunth. In: Barkworth ME Capels KM Long S Piep MB (Eds) Magnoliophyta: Commelinidae (in part): Poaceae, part 2 Flora of North America North of Mexico, Vol 25. Oxford University Press, New York, 200–203.

[B21] Rzedowski J (1959) Las colecciones botanicas de Wilhelm (Jose Guillermo) Schaffner en San Luis Potosí. I. Acta Científica Potosina 3: 99–121.

[B22] Soreng RJ Peterson PM Romaschenko K Davidse G Teisher JK Clark LG Barberá P Gillespie LJ Zuloaga FO (2017) A worldwide phylogenetic classification of the Poaceae (Gramineae) II: An update and a comparison of two 2015 classifications. Journal of Systematics and Evolution 55(4): 259–290. http://onlinelibrary.wiley.com/doi/10.1111/jse.12262/full 10.1111/jse.12262

[B23] Watson L Dallwitz M (1992) The Grass Genera of the World. CAB International, Wallingford, 1038 pp.

